# Liver cirrhosis: relationship between fibrosis-associated hepatic morphological changes and portal hemodynamics using four-dimensional flow magnetic resonance imaging

**DOI:** 10.1007/s11604-023-01388-0

**Published:** 2023-01-19

**Authors:** Atsushi Higaki, Akihiko Kanki, Akira Yamamoto, Yu Ueda, Kazunori Moriya, Hiroyasu Sanai, Hidemitsu Sotozono, Tsutomu Tamada

**Affiliations:** 1 Department of Radiology, Kawasaki Medical School, 577 Matsushima, Kurashiki City, Okayama 710-0192 Japan; 2 Philips Japan, 2-13-37 Konan, Minato-Ku, Tokyo, 108-8507 Japan

**Keywords:** Four-dimensional flow magnetic resonance imaging, Hepatic morphology, Liver cirrhosis, Portal hemodynamics

## Abstract

**Purpose:**

The mechanisms underlying the morphological changes in liver cirrhosis remain unknown. This study aimed to clarify the relationship between fibrotic hepatic morphology and portal hemodynamic changes using four-dimensional flow magnetic resonance imaging (MRI).

**Materials and methods:**

Overall, 100 patients with suspected liver disease who underwent 3-T MRI were evaluated in this retrospective study. Liver fibrosis was assessed using a combination of visual assessment of the hepatic morphology and quantitative measures, including the fibrosis-4 index and aspartate transaminase-to-platelet ratio. It was classified into three groups according to the severity of fibrosis as follows: A (normal), B (mild-to-moderate), and C (severe). Quantitative indices, including area (mm^2^), net flow (mL/s), and average velocity (cm/s), were measured in the right portal vein (RPV) and left portal vein (LPV), and were compared across the groups using the Kruskal–Wallis and Mann–Whitney *U* tests.

**Results:**

Among the 100 patients (69.1 ± 12.1 years; 59 men), 45, 35, and 20 were categorized into groups A, B, and C, respectively. The RPV area significantly differed among the groups (from *p* < 0.001 to *p* = 0.001), showing a gradual decrease with fibrosis progression. Moreover, the net flow significantly differed between groups A and B and between groups A and C (*p* < 0.001 and *p* < 0.001, respectively), showing a decrease during the early stage of fibrosis. In the LPV, the net flow significantly differed among the groups (from *p* = 0.001 to *p* = 0.030), revealing a gradual increase with fibrosis progression.

**Conclusion:**

The atrophy–hypertrophy complex, which is a characteristic imaging finding in advanced cirrhosis, was closely associated with decreased RPV flow in the early stage of fibrosis and a gradual increase in LPV flow across all stages of fibrosis progression.

## Introduction

Chronic liver disease is characterized by progressive fibrosis of the liver parenchyma due to persistent inflammation, damage, and circulatory disturbances, ultimately leading to cirrhosis. Therefore, hepatic fibrosis progression and hemodynamic changes in the portal system are essential prognostic indicators of chronic liver disease [1, 2]. The compensatory stage of cirrhosis shows characteristic selective atrophy and compensatory hypertrophy (i.e., the atrophy–hypertrophy complex) [3–6]. On histopathological examination, liver fibrosis is associated with reduced circulating blood volume in the sinusoids because of short-circuiting of the microvessels, crushing of portal vein (PV) branches and hepatic veins due to compression by regenerative nodules, and disorganization of the hepatocellular cord-arrangement [7].

Morphological changes in the liver are heterogeneous, with atrophy occurring in the right lobe and hypertrophy in the left and caudate lobes. These morphological changes are related to the anatomy of the PV and hepatic vein, the distribution of hormones, nutritional factors, and inflammatory mediators in the liver [8–12]. Particularly, the uneven distribution of macroscopic blood flow due to anatomical differences between the right portal vein (RPV) and left portal vein (LPV) is assumed to be closely related to changes in the liver morphology in chronic liver disease; however, no studies have confirmed this mechanism.

Although computed tomography (CT) and magnetic resonance imaging (MRI) can anatomically identify the development of collateral circulation, early portal hemodynamic changes cannot be assessed [13]. Doppler ultrasonography (US) remains the first choice for evaluating blood flow in the PV system. However, it has many limitations, including the inability to observe deep areas and many sites simultaneously, the narrow field-of-view (FOV), the possibility of different measurements depending on the angle, and poor interobserver reproducibility [14–16].

Three-dimensional (3D) cine (continuous) phase-contrast MRI, known as four-dimensional (4D) flow MRI, has recently been developed to enable comprehensive and retrospective assessment of the entire upper abdominal hemodynamics by simultaneously and noninvasively acquiring time-resolved flow and anatomical information in a 3D imaging volume [15, 16]. This technology enables visualization of cine (continuous) images reflecting hemodynamics in 3D from any direction, calculating the flow velocity and rate in any cross-section, vector information in blood vessels, and shear stress. In the portal system, several studies on cirrhosis and its complications have been reported, as well as on treatment planning and follow-up for liver surgery and intervention [17–30].

This study aimed to clarify the relationship between liver fibrosis-associated hepatic morphology and portal hemodynamic changes, assessed using 4D flow MRI.

## Materials and methods

### Design and participants

This single-center retrospective study was conducted in accordance with the principles embodied in the Declaration of Helsinki and was approved by the Institutional Review Board of Kawasaki Medical School (approval number: 3949-01). The requirement for informed consent was waived because of the retrospective study design. All methods were performed in accordance with the relevant guidelines and regulations.

**Fig. 1:**
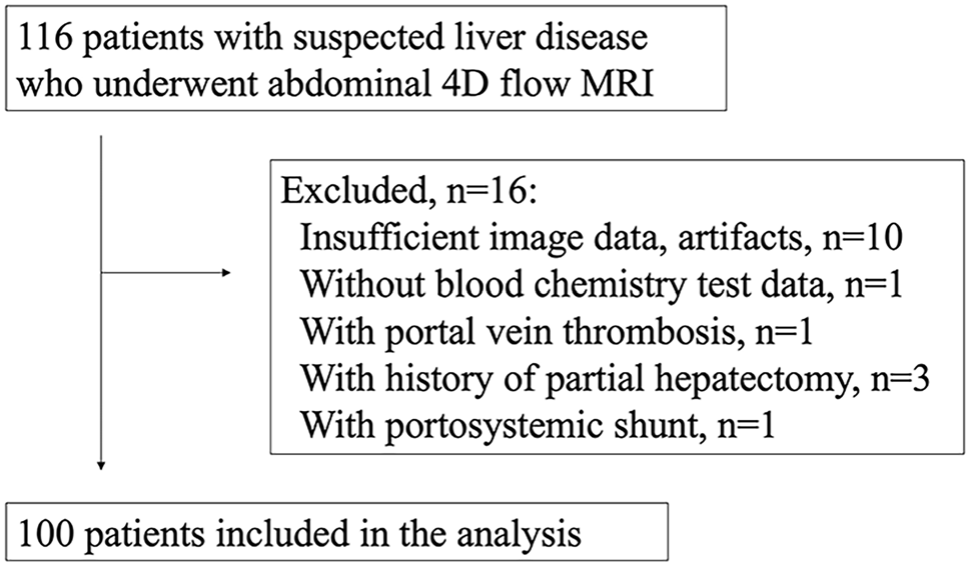
Patient inclusion flowchart. Abbreviations: *4D* four-dimensional, *MRI* magnetic resonance imaging

We evaluated 116 consecutive patients with suspected liver disease who underwent abdominal MRI, including 4D flow MRI at our hospital from November 2019 to May 2021. Sixteen patients were excluded due to poor imaging quality or missing data (*n* = 10); we further excluded patients with a congenital portal shunt (*n* = 1), partial hepatectomy (*n* = 3), PV thrombosis (*n* = 1), and no blood reports (*n* = 1). Therefore, 100 patients (69.1 ± 12.1 years; 59 men) were enrolled (Fig. 1).

### MRI technique

All MRI examinations were performed with a 3-T scanner using a 32-channel phased-array coil (anterior and posterior coil; Ingenia Elition 3.0 T or Ingenia 3.0 T CX Quasar Dual; Philips Healthcare, Best, Netherlands); patients fasted for ≥ 3 h before the examination [27]. A 0.1-mL/kg dose of gadoxetic acid (Primovist; Bayer Schering Pharma, Berlin, Germany; 0.025 mmol/kg) was administered during clinical MRI, and portal venous and hepatobiliary phase images were obtained at 70 s and 20 min after contrast material injection, respectively. In the hepatobiliary phase, the imaging parameters for 3D T1-weighted gradient-echo sequences with fat suppression were as follows: repetition time (TR), 4.0 ms; time to echo (TE), 1.43 ms; flip angle, 20°; bandwidth, 1157.4 Hz; parallel imaging factor, 2 (phase) × 1 (slice); FOV, 350 × 295 mm^2^; voxel size, 1.22 × 1.53 × 5.00 mm^3^ (reconstructed to 0.68 × 0.68 × 2.50 mm^3^ with interpolation) without the combination of the k-space shutter; and acquisition time, 18.4 s.

We performed 4D flow MRI with 4D velocity maps acquired using phase-contrast imaging of coronal or coronal-oblique 120-mm slabs. This was tailored to the individual’s anatomy to provide comprehensive coverage of the upper abdominal vessels. The 4D flow MRI parameters were as follows: FOV, 400 × 400 mm^2^; TR/TE, 5.2–5.3 ms; TE, 3.6–3.7 ms; sensitivity encoding factor, 3.5 (phase × slice = 3.5 × 1.0); flip angle, 10°; voxel size, 1.67 × 2.38 × 4.00 mm^3^ (reconstructed to 0.83 × 0.83 × 2.00 mm^3^ with interpolation) without the combination of the k-space shutter; and velocity-encoding sensitivity (VENC), 20 cm/s in all three flow encoding directions (RL-AP-FH). The approximate scan time for 4D Flow MRI was 8–20 min. Images were obtained under free-breathing conditions to minimize respiratory-induced motion artefacts using a navigator-gated approach. Diaphragmatic synchronization was performed with a navigator length of 80 mm positioned on the lung-liver interface, and only data from the middle 6 mm were used. The approximate acceptance ratio was 30–75%. Pulse wave synchronization was used to synchronize the data acquisition with the cardiac cycle; data were retrospectively classified into 10 cardiac time phases. Finally, all three components of the phase-contrast angiogram and velocity vector field were generated using an offline reconstruction.

### Image analysis and data collection

To evaluate fibrosis-associated hepatic morphological changes, morphological severity of cirrhosis and clinical data, including the fibrosis-4 (Fib-4) index and aspartate transaminase-to-platelet ratio index (APRI), were used. MRI assessment was performed using a standard picture archiving and communication system (SYNAPSE; Fujifilm, Tokyo, Japan). Regarding the grade of the morphological severity of cirrhosis, two fellowship-trained radiologists with 15 and 23 years of experience in abdominal MRI (A.H. and A.Y., respectively), who were blinded to the patients’ clinical data, independently performed the image analyses. Disagreements were adjudicated by a third fellowship-trained radiologist with 16 years of experience in abdominal MRI (A.K.).

**Fig. 2:**
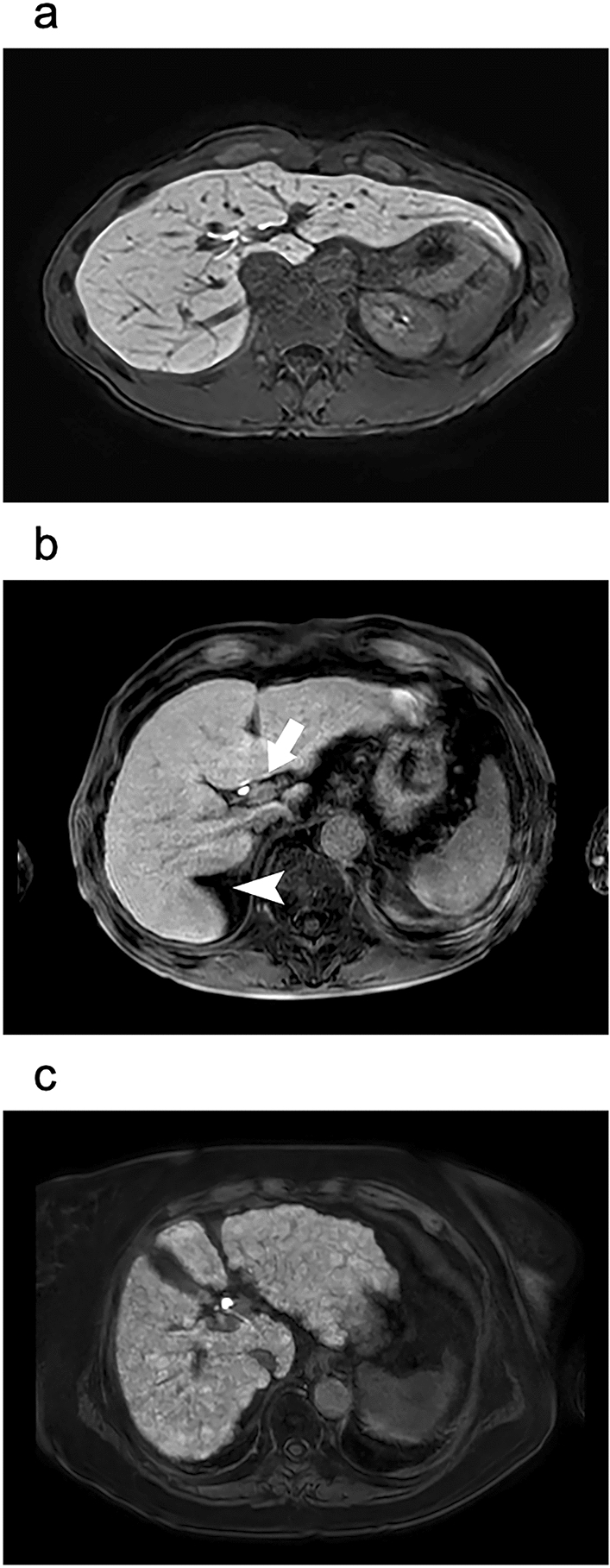
Assessment of the morphological severity of cirrhosis using hepatobiliary phase images in gadoxetic acid-enhanced MRI. **a** Group A (no cirrhosis): hepatobiliary phase imaging in a 50-year-old male patient showing no morphological changes related to cirrhosis. **b** Group B (mild-to-moderate cirrhosis): a hepatobiliary phase image of a 71-year-old male patient showing enlargement of the hilar periportal space (arrow) and right posterior hepatic notch sign (arrowhead). **c** Group C (severe cirrhosis): a hepatobiliary phase image of an 81-year-old male patient showing a nodular surface of the liver caused by regenerative nodules. Abbreviation: *MRI* magnetic resonance imaging

The morphological grades of cirrhosis were categorized into group A (no cirrhosis), including those without morphological changes related to cirrhosis (Fig. 2a); group B (mild-to-moderate cirrhosis), including those with enlarged hilar periportal space and/or expanded gallbladder fossa sign and/or right posterior hepatic notch sign (Fig. 2b); and group C (severe cirrhosis), with nodularity of the liver surface and/or visible regenerative nodules combined with an expanded gallbladder fossa sign and/or right posterior hepatic notch sign (Fig. 2c) [5, 6, 12, 31].

The presence of splenomegaly and collateral circulation was recorded. The final fibrosis-associated hepatic morphological grade was determined by adding the threshold values of the Fib-4 index and APRI to the morphological grades of cirrhosis severity [32–35]. Group A was upgraded to group B if the Fib-4 index was > 1.3 and the APRI was > 0.5, whereas group B was upgraded to group C if the Fib-4 index was > 2.67 and the APRI was > 1. Group B was downgraded to group A if the Fib-4 index was ≤ 1.3 and the APRI was ≤ 0.5, whereas group C was downgraded to group B if the Fib-4 index was ≤ 2.67 and the APRI was ≤ 1.

**Fig. 3:**
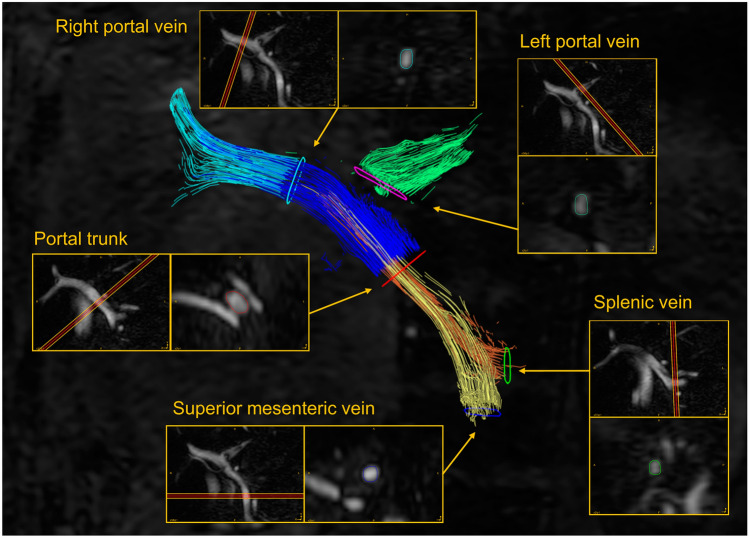
Velocity map of four-dimensional (4D) flow MRI of a 46-year-old female patient with normal liver (Fib-4 index = 0.39, APRI = 0.16). The 4D flow MRI velocity map shows the flow in the portal trunk (PT, dark blue), right portal vein (RPV, light blue), left portal vein (LPV, green), superior mesenteric vein (SMV, yellow), and splenic vein (SV, orange). For 4D blood flow analysis, the radiologists manually defined the region of interest on the PT, RPV, LPV, SMV, and SV cutting planes. Abbreviations: *APRI* aspartate aminotransferase-to-platelet ratio index, *Fib-4 index* fibrosis-4 index, *MRI* magnetic resonance imaging

For the quantitative evaluation of 4D flow MRI, blood flow was analyzed using GTFlow (GyroTools LLC, Zurich, Switzerland). In addition to hemodynamic visualization, the same radiologists (A.H. and A.Y.) manually placed a region of interest in consensus on the cutting planes of the portal trunk (PT), RPV, LPV, superior mesenteric vein (SMV), and splenic vein (SV). To assess the accuracy of the placement of regions of interest on PCA images, we compared the area of PCA with that of contrast-enhanced MR angiography on portal phase images and found a significant correlation in all planes (Spearman’s *ρ* = 0.888 to 0.966, *p* < 0.001). Therefore, we manually set up regions of interest in the PCA images. Subsequently, the area (vessel diameter, mm^2^), forward flow (mL/s), backward flow (mL/s), net flow (mL/s; the difference between forward and backward flows), total flow (mL/s; the sum of forward and backward flows), and average velocity (cm/s) in each cross-section were calculated (Fig. 3). All vessel segmentation was performed manually without using a threshold. Quantitative values were calculated as averages of data obtained using time-varying (time-resolving) PCA, and the approximate analysis time was 5–10 min per case.

**Fig. 4:**
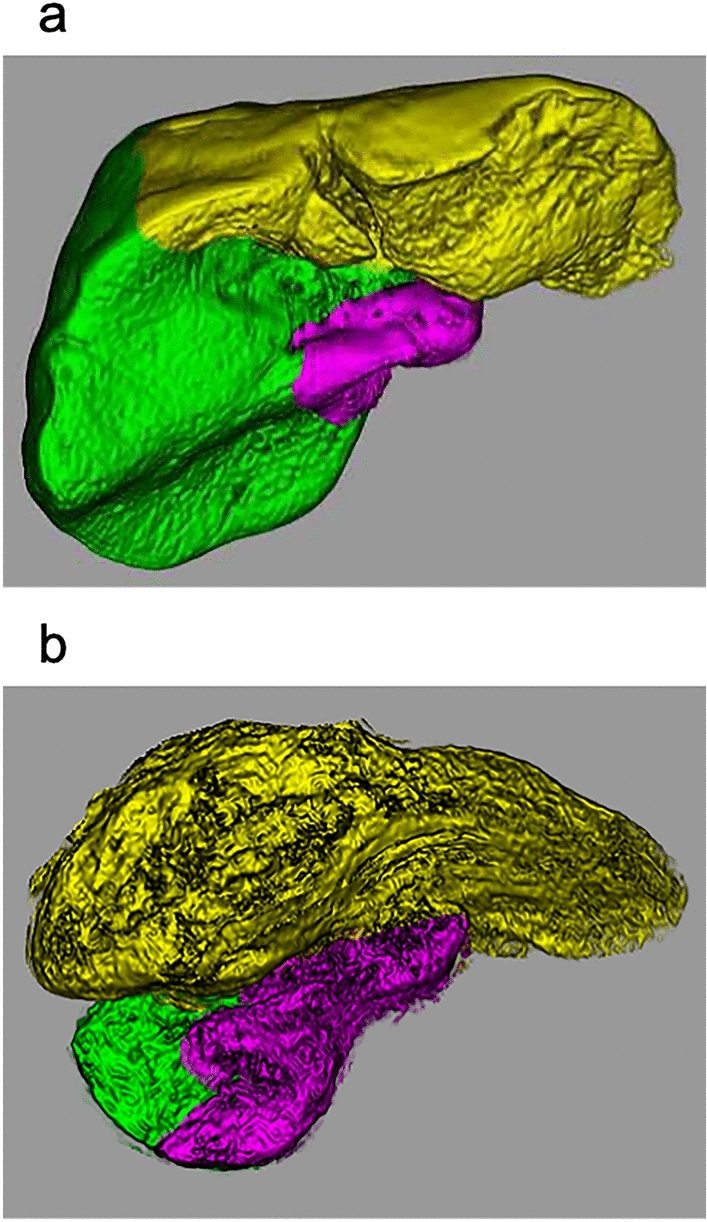
Liver segmentation and volume measurements. Three-dimensional (3D) volume data of the liver in representative cases of **a** group A (a 41-year-old female patient with a normal liver, right lobe = 971.0 mL, left lobe = 544.1 mL, and caudate lobe = 29.1 mL) and **b** group C (a 69-year-old female patient with liver cirrhosis, right lobe = 249.8 mL, left lobe = 1630.8 mL, and caudate lobe = 83.5 mL). The liver image was extracted semi-automatically from the hepatobiliary phase images in gadoxetic acid-enhanced magnetic resonance imaging and then manually segmented. The volume of each region was calculated using 3D simulation software. The green, yellow, and pink regions indicate the right, left, and caudate lobes, respectively

Liver volumes were measured using a 3D simulation software (SYNAPSE VINCENT; Fujifilm, Tokyo, Japan). Briefly, the liver image was semi-automatically extracted from the hepatobiliary phase images. Next, the radiologist (A.H.) manually separated the blood vessels, bile ducts, and perihepatic soft tissues and then determined the right, left, and caudate lobes based on the right hepatic and middle hepatic veins and country lines [36]. Subsequently, the volume (mL) of each region was calculated (Fig. 4). Interobserver reliability was assessed with another radiologist (H.S.) using Spearman’s rank correlation coefficient. The Spearman's correlation coefficients (*ρ*) of the right, left, and caudate lobes were 0.959, 0.962, and 0.937, respectively. These results showed an excellent agreement between the observers. Therefore, we used the liver volumes measured by the first radiologist.

### Statistical analyses

The Kruskal–Wallis test was performed to assess significant differences in quantitative indices of the PT, RPV, LPV, SMV, and SV, as well as the liver volumes (right, left, and caudate lobes) among the three groups. If significant differences were found, pairwise comparisons were performed using the Mann–Whitney *U* test. The relationship between the right lobe volume and net flow in the RPV, left lobe volume and net flow in the LPV, and caudate lobe volume and net flow in the LPV was assessed using Spearman’s rank correlation coefficient (*ρ*). All statistical tests were performed using SPSS Statistics version 24 (IBM Corp., Armonk, NY, USA), and a two-sided *p* value < 0.05 was considered statistically significant.

## Results

### Patient characteristics

Overall, 67 patients had chronic liver disease. Of these, 36 and 31 patients did and did not have cirrhosis, respectively. Among patients with cirrhosis, 29, 6, and 1 had Child − Pugh class A, B, and C disease, respectively. Table 1 shows the patient characteristics. Fifteen patients (15%) had no focal liver lesions on MRI. Among the remaining 85 patients (85%), 23, 2, 1, 3, 5, 3, 14, 15, and 19 had hepatocellular carcinoma, cholangiocarcinoma, combined hepatocellular-cholangiocarcinoma, dysplastic nodules, hyperplastic nodules, liver abscess, liver cysts, hepatic hemangioma, and metastatic liver tumors (1, pancreatic cancer; 1, extrahepatic cholangiocarcinoma; 1, gallbladder cancer; 1, myxofibrosarcoma; 3, gastrointestinal stromal tumor; 1, adrenal cancer; and 11, colorectal cancer), respectively.

**Table 1:** Patient characteristics by group

Characteristic	Group A	Group B	Group C
Number of patients	45	35	20
Age, years	64.3 ± 14.2	71.8 ± 8.0	75.1 ± 8.40
Sex (male/female)	23/22	23/12	13/7
Presence and etiology of chronic liver disease			
Patients without chronic liver disease	33 (73)	0 (0)	0 (0)
Patients with chronic liver disease	12 (27)	35 (100)	20 (100)
Presence of cirrhosis	0 (0)	18 (51)	18 (90)
HBV	2	4	1
HCV	3	10	3
Alcohol	1	7	5
NAFLD/NASH	3	5	3
Primary biliary cholangitis	1	0	0
HBV and NAFLD/NASH	1	0	0
HCV and NAFLD/NASH	0	1	0
HBV and alcohol	0	1	1
HCV and alcohol	0	1	2
Autoimmune hepatitis and NAFLD/NASH	0	0	1
Hemochromatosis and alcohol	0	0	1
Cryptogenic	1	6	3
Scoring systems in liver fibrosis			
Fib-4 index	1.38 ± 0.61	2.52 ± 1.03	6.14 ± 2.63
APRI	0.3 ± 0.1	0.52 ± 0.2	1.57 ± 0.9
Others			
Patients with collateral vessels	0 (0)	3 (9)	15 (75)
Patients with splenomegaly	1 (2)	3 (9)	11 (55)

Data are presented as *n* (%) or mean ± standard deviation

Groups A, B, and C included patients without cirrhosis, with mild-to-moderate cirrhosis, with severe cirrhosis, respectively

*APRI* aspartate aminotransferase-to-platelet ratio index, *Fib-4 index* fibrosis-4 index, *HBV* hepatitis B virus, *HCV* hepatitis C virus, *NAFLD* non-alcoholic fatty liver disease, *NASH* non-alcoholic steatohepatitis

Overall, 45 patients were categorized into group A (no cirrhosis, mean age, 64 [23–92] years; 23 men and 22 women); 35 into group B (mild-to-moderate cirrhosis, mean age, 72 [53–89] years; 23 men and 12 women); and 20 into group C (severe cirrhosis, mean age, 75 [53–91] years; 13 men and seven women) (Table 1).

The percentages of patients with collateral vessels were 0/45 (0%), 3/35 (9%), and 15/20 (75%) in groups A, B, and C, respectively. However, we did not find any shunts to the paraumbilical vein that would increase blood flow in the LPV or to the right adrenal or intercostal veins that would increase blood flow in the RPV.

### Quantitative analysis of portal hemodynamics and liver volume

The three groups differed with respect to backward flow and average velocity in the PT; area and average velocity in the SMV; area, total flow, and forward flow in the SV; area, net flow, total flow, forward blood flow, and backward flow in the RPV; net flow, total flow, and forward flow in the LPV; right lobe volume; left lobe volume; and caudate lobe volume (Table 2).

**Table 2:** Quantitative analysis of four-dimensional flow magnetic resonance imaging

Parameter and structure	Index	Group A	Group B	Group C	*p* value^*^	*p* value^‡^			Parameter and structure	Index	Group A	Group B	Group C	*p* value^*^	*p* value^‡^		
						GroupA vs B	GroupA vs C	GroupB vs C							GroupA vs B	GroupA vs C	GroupB vs C
Portal trunk	Area (mm^2^)	73.2 ± 24.3	78.1 ± 26.6	70.4 ± 31.2	0.63*	N/A	N/A	N/A	Right portal vein	Area (mm^2^)	72.6 ± 16.8^‡§^	58.0 ± 18.9	41.7 ± 11.6	< 0.001^†^	0.001^§^	< 0.001^§^	0.001^§^
	Net flow (mL/s)	7.03 ± 2.80	5.94 ± 3.46	6.28 ± 3.08	0.19*	N/A	N/A	N/A		Net flow (mL/s)	5.02 ± 1.76^‡§^	3.51 ± 1.59	2.94 ± 1.63	< 0.001^†^	< 0.001^§^	< 0.001^§^	0.142^‡^
	Total flow (mL/s)	7.51 ± 2.75	6.97 ± 3.05	6.31 ± 3.08	0.36*	N/A	N/A	N/A		Total flow (mL/s)	5.42 ± 1.79^‡§^	3.91 ± 1.60	2.98 ± 1.62	< 0.001^†^	< 0.001^§^	< 0.001^§^	0.031^§^
	Forward flow (mL/s)	7.27 ± 2.76	6.46 ± 3.21	6.30 ± 3.08	0.25*	N/A	N/A	N/A		Forward flow (mL/s)	5.22 ± 1.76^‡§^	3.71 ± 1.57	2.96 ± 1.62	< 0.001^†^	< 0.001^§^	< 0.001^§^	0.06^§^
	Backward flow (mL/s)	0.24 ± 0.32^§^	0.51 ± 0.57	0.02 ± 0.03	< 0.001^†^	0.24^‡^	< 0.001^§^	< 0.001^§^		Backward flow (mL/s)	0.20 ± 0.24^§^	0.20 ± 0.29	0.02 ± 0.05	< 0.001^†^	0.312^‡^	< 0.001^§^	0.02^§^
	Average velocity (cm/s)	12.1 ± 1.76^*†*^	11.04 ± 2.16	9.53 ± 2.16	0.001^†^	0.244^‡^	< 0.001^§^	< 0.001^§^		Average velocity (cm/s)	9.73 ± 2.49	8.98 ± 2.35	8.53 ± 3.06	0.21*	N/A	N/A	N/A
Superior mesenteric vein	Area (mm^2^)	47.5 ± 16.5^§^	50.5 ± 16.5	65.1 ± 28.2	0.013^†^	0.335^‡^	0.004^§^	0.034^§^	Left portal vein	Area (mm^2^)	35.1 ± 14.1	52.8 ± 52.8	46.2 ± 21.5	0.14*	N/A	N/A	N/A
	Net flow (mL/s)	3.62 ± 1.45	3.98 ± 1.65	4.16 ± 1.73	0.60*	N/A	N/A	N/A		Net flow (mL/s)	1.63 ± 0.89^‡§^	2.94 ± 2.71	3.10 ± 1.73	0.001^†^	0.004^§^	0.001^§^	0.029^§^
	Total flow (mL/s)	3.77 ± 1.36	4.20 ± 1.87	4.21 ± 1.72	0.71*	N/A	N/A	N/A		Total flow (mL/s)	1.87 ± 1.03^‡§^	3.18 ± 2.73	3.15 ± 1.77	0.004^†^	0.009^§^	0.004^§^	0.565^‡^
	Forward flow (mL/s)	3.69 ± 1.39	4.09 ± 1.74	4.18 ± 1.72	0.66*	N/A	N/A	N/A		Forward flow (mL/s)	1.75 ± 0.92^‡§^	3.01 ± 2.74	3.13 ± 1.75	0.003^†^	0.001^§^	0.002^§^	0.404^‡^
	Backward flow (mL/s)	0.08 ± 0.17	0.11 ± 0.29	0.02 ± 0.06	0.41*	N/A	N/A	N/A		Backward flow (mL/s)	0.12 ± 0.28	0.12 ± 0.30	0.03 ± 0.06	0.39*	N/A	N/A	N/A
	Average velocity (cm/s)	9.13 ± 2.27^§^	8.93 ± 2.05^¶^	7.11 ± 1.55	0.001^†^	0.513^‡^	0.002^§^	0.004^§^		Average velocity (cm/s)	7.10 ± 2.30	8.94 ± 8.09	7.69 ± 2.41	0.49*	N/A	N/A	N/A
Splenic vein	Area (mm^2^)	33.8 ± 9.25^§^	33.6 ± 12.4^¶^	44.2 ± 14.9	0.005^†^	0.825	0.002^§^	0.004^§^	Right lobe	Volume (mL)	910 ± 205^§^	835 ± 266	599 ± 252	< 0.001^†^	0.189^‡^	0.001^§^	0.006^§^
	Net flow (mL/s)	2.03 ± 1.09	1.92 ± 1.13	2.54 ± 1.84	0.08*	N/A	N/A	N/A	Left lobe	Volume (mL)	412 ± 163^‡§^	485 ± 140	646 ± 344	< 0.001^†^	0.006^§^	0.001^§^	0.077^‡^
	Total flow (mL/s)	2.39 ± 0.94^§^	2.32 ± 1.25^¶^	3.35 ± 1.15	0.003^†^	0.5^‡^	0.002^§^	0.002^§^	Caudate lobe	Volume (mL)	32.5 ± 16.5^§^	33.1 ± 17.8	54.8 ± 23.7	< 0.001^†^	0.942^‡^	< 0.001^§^	0.001^§^
	Forward flow (mL/s)	2.21 ± 0.98^§^	2.12 ± 1.15^¶^	2.95 ± 1.33	0.031^†^	0.487^‡^	0.026^§^	0.015^§^									
	Backward flow (mL/s)	0.18 ± 0.28	0.20 ± 0.31	0.41 ± 0.76	0.93*	N/A	N/A	N/A									
	Average velocity (cm/s)	9.14 ± 2.04	8.39 ± 2.39	9.58 ± 2.83	0.21*	N/A	N/A	N/A									

Group A included patients without cirrhosis, group B included patients with mild-to-moderate cirrhosis, and group C included patients with severe cirrhosis

^*^Kruskal–Wallis test among the three groups

^†^Kruskal–Wallis test; significant difference with *p* < 0.05

^‡^Mann–Whitney *U* test for pairwise comparisons

^§^Mann–Whitney test; significant difference with *p* < 0.05

*N/A* not applicable

In the PT, the backward flow and average velocity differed between groups A and C and between groups B and C (Table 2). However, in the SMV, the area and average velocity differed between groups A and C and between groups B and C (Table 2). In the SV, area, total flow, and forward flow differed between groups A and C and between groups B and C (Table 2). In the RPV, area, total flow, and forward blood flow differed between groups A and B, between groups A and C, and between groups B and C (Table 2). The net flow differed between groups A and B and between groups A and C. The backward flow differed between groups A and C and between groups B and C (Table 2). In the LPV, net flow differed between groups A and B, between groups A and C, and between groups B and C; total flow and forward flow differed between groups A and B (*p* = 0.009 and *p* = 0.01, respectively) and between groups A and C (Table 2).

**Fig. 5:**
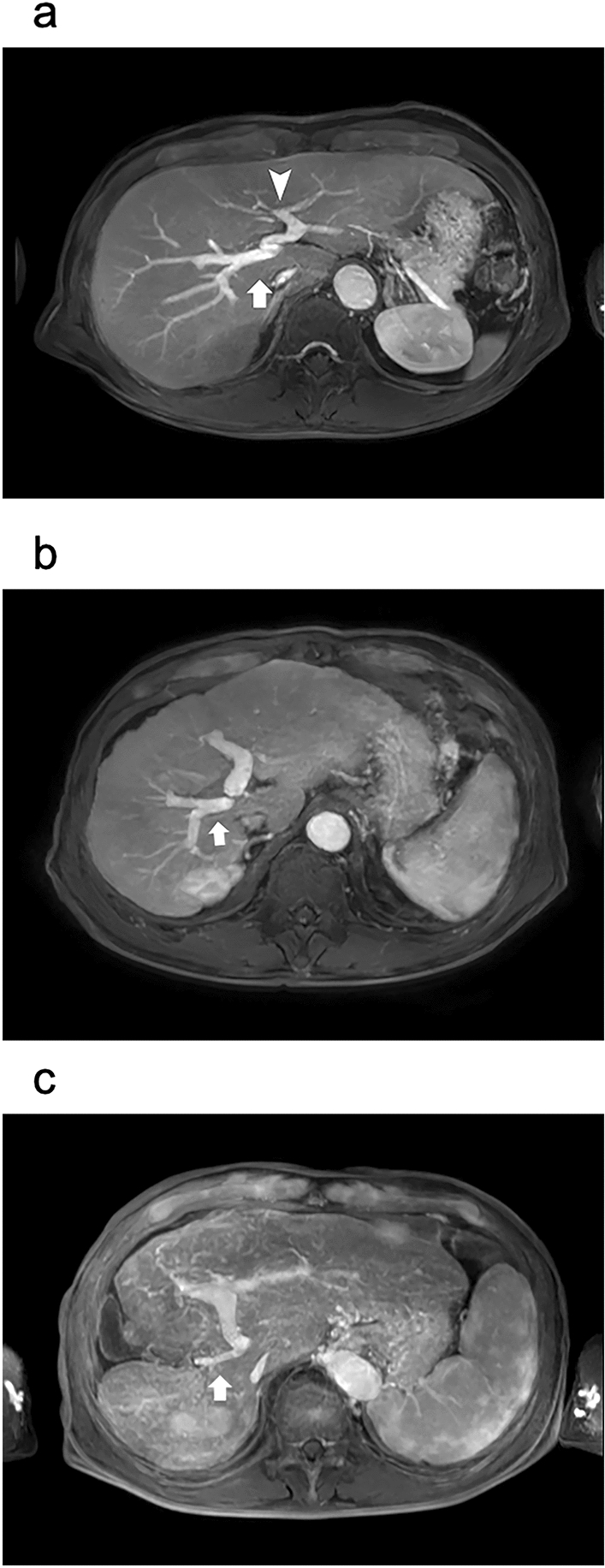
Anatomical differences between the right portal vein (RPV) and left portal vein (LPV) in normal liver and fibrotic liver. Serial changes in the vascular diameter and liver volume in the RPV and LPV can be observed with liver fibrosis (portal phase images in gadoxetic acid-enhanced MRI). **a** In group A (no cirrhosis), the RPV branches linearly from the portal trunk (PT) and is immediately surrounded by liver parenchyma (arrow). The LPV branches tortuously from the PT and is surrounded by the hepatic parenchyma via the falciform ligament (arrowhead). **b** In group B (mild-to-moderate cirrhosis), the RPV (arrow) is thin, and the left lobe is enlarged. **c** In group C (severe cirrhosis), the liver with severe cirrhosis shows further thinning of the RPV (arrow), marked atrophy of the right lobe, and marked enlargement of the left and caudate lobes. Abbreviations: *LPV* left portal vein, *RPV* right portal vein

For liver volume, that of the right lobe differed between groups A and C and between groups B and C. The left lobe volume differed significantly between groups A and B (*p* = 0.006) and between groups A and C; the caudate lobe volume differed between groups A and C and between groups B and C (Table 2, Fig. 5).

**Fig. 6:**
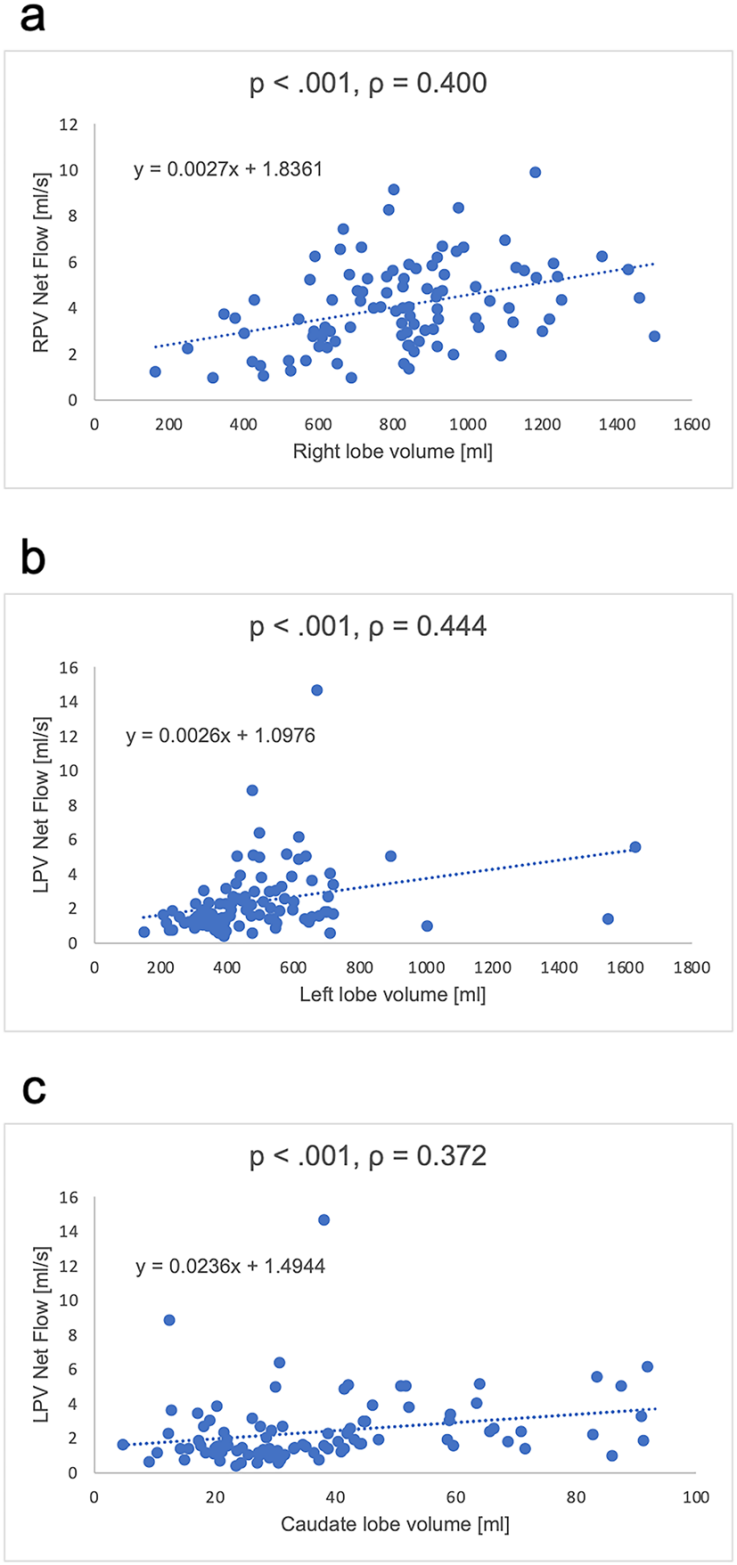
Graph showing the correlation between the net flow and liver volume. The scatter plot shows positive correlations between **a** RPV net flow and right lobe volume, **b** LPV net flow and left lobe volume, and **c** LPV net flow and caudate lobe volume. Abbreviations: *LPV* left portal vein, *RPV* right portal vein

Regarding the correlation of the net flows in the RPV and LPV with liver volumes (right, left, and caudate lobes), the net flow in the RPV positively correlated with the right lobe volume. Similarly, the net flow in the LPV positively correlated with the left lobe and caudate lobe volumes (Fig. 6).

## Discussion

In this study, fibrosis-associated hepatic morphological changes were strongly correlated with the indices of portal hemodynamics and liver volume. First, the net flow in the RPV showed a significant positive correlation with right lobe volume. Additionally, the net flow in the LPV showed a significantly positive correlation with the left and caudate lobe volumes. These findings indicate that characteristic imaging findings of advanced cirrhosis, including right lobe atrophy and left and caudate lobe enlargement, may be associated with blood flow changes in the PV.

Second, net flow in the RPV decreased in the early stages of liver fibrosis: area, total flow, and forward flow gradually decreased with fibrosis progression. However, backward flow decreased in the advanced stages of liver fibrosis. In the LPV, forward and total flow increased in the early stages of liver fibrosis: net flow gradually increased with liver fibrosis. These changes contributed to the obstruction of the RPV due to liver fibrosis, leading to a compensatory increase in the blood flow in the LPV. RPV is more likely to be affected by fibrotic obstruction than the LPV, because it bifurcates linearly from the PT and is immediately surrounded by liver parenchyma.

Additionally, the LPV bends and branches from the PT while surrounded by hepatic parenchyma via a long and tortuous path through the falciform ligament [8, 13]. Therefore, the RPV may be more susceptible to increased vascular resistance than the LPV due to liver fibrosis. Consequently, when fibrosis progresses from a mild-to-moderate degree, net flow in the RPV first reduces, followed by right lobe atrophy. However, subsequent compensatory elevation in blood flow in the LPV may lead to left and caudate lobe hypertrophy. The caudate lobe is supplied by both the RPV and LPV, but receives more blood from the LPV. Therefore, the weak correlation of net flow in the LPV with caudate lobe volume (*ρ* = 0.372) than with left lobe volume (*ρ* = 0.444) may indicate the influence of double blood supply in the caudate lobe.

Third, as fibrosis progressed, the net flow in the RPV decreased in the early stage, followed by a decrease in the right lobe volume. In contrast, the forward flow in the LPV increased as liver fibrosis progressed, and this occurred almost simultaneously with an increase in the left lobe volume. These results suggest that changes in liver volume were affected more rapidly with increased than with decreased portal blood flow. In patients with severe liver fibrosis, a marked increase in vascular resistance prevents portal blood flow into the liver, resulting in the dilatation of anastomotic branches to the veins and formation of extrahepatic collateral circulation [37]. These collateral pathways are crucial, because they reduce the congestion of the portal blood flow and portal pressure; however, they further cause severe clinical syndromes such as gastrointestinal varices, hepatic encephalopathy, ascites, splenomegaly, and hypersplenism [38, 39].

Here, backward flow and average velocity in the PT were decreased in advanced stages of liver fibrosis, as indicated by significant differences between groups A and C and between groups B and C. This occurrence was mainly due to the formation of a gastrorenal shunt that allowed backward flow into the left gastric vein branching from the main trunk of the PV and the development of extrahepatic collateral circulation, reducing the flow into the PV [19, 37]. Several previous studies evaluated the relationship between liver fibrosis and PV hemodynamics with ultrasound (US) and reported that the flow velocity significantly decreased as cirrhosis became more severe [40–42]. Although 4D flow MRI tends to observe lower mean flow velocities than the US, both measurements reportedly have a good agreement and an excellent correlation [24, 43, 44]. Our results using 4D flow MRI also showed a significant decrease in portal venous flow velocity with more severe liver fibrosis, consistent with previous studies. Flow measurement using the US is simpler and less expensive than MRI, but 4D flow MRI is advantageous, because it is more objective and reliable since it measures perpendicularly to the vessel and allows retrospective and simultaneous evaluations of multiple vessels with strong repeatability and reproducibility, as well as internal consistency [17, 25, 45, 46]. We speculated that the increase in area and decrease in average velocity in the SMV, in addition to the increase in area, total flow, and forward flow in the SV in advanced stages of liver fibrosis, resulted from the development of collateral circulation, mainly splenic and renal shunts, and hyperdynamic syndrome [19, 37, 47].

In a previous report evaluating hemodynamics in healthy individuals and patients with cirrhosis using 4D flow MRI, patients with cirrhosis exhibited a decreased average velocity in the PT, an increased area, net flow in the LPV and SV, an increased area in the SMV, and a decrease in the average velocity [24]. In patients with cirrhosis, the LPV and RPV areas did not differ, whereas the area of LPV was smaller than that of RPV in healthy individuals. These results are consistent with those of this study. In a previous study, the area and net flow in the RPV were also reduced in patients with cirrhosis, as in this study. However, no significant difference was found between the two groups, possibly due to the smaller sample size (20 patients with cirrhosis vs. 41 healthy patients) than in this study. In summary, the hemodynamic changes in the left and right portal veins associated with cirrhosis and those in the SV and SMV may be due to hyperdynamic syndrome, which is consistent with this study’s results. The novelty of this study, in addition to these results, is that it differentiated between normal, early, and late fibrotic livers and showed that portal hemodynamic and hepatic volume changes occur gradually and are related to the atrophy–hypertrophy complex, which is a characteristic imaging finding in cirrhosis.

This study has some limitations. First, it was a retrospective, single-center, cross-sectional study with a limited number of patients. Second, since we did not have a sub-cohort of sufficient size with histopathological confirmation, changes in fibrosis-associated hepatic morphology were assessed using a combination of visual assessment and quantitative measures. However, imaging techniques, such as MRI, and non-invasive markers, such as the Fib-4 index and APRI, have relatively good accuracy for assessing hepatic fibrosis [1, 37, 47–49]. Furthermore, we believe that these assessments are reliable indicators, because compared with the local assessment of a biopsy, they allow the evaluation of the entire liver. Third, this study included various etiologies of chronic liver disease; therefore, the atrophy–hypertrophy complex in the compensatory stage of cirrhosis may vary depending on the etiology. Fourth, we did not evaluate arterial blood flow, because we used VENC targeting the portal system. Because of the hepatic buffer effect, arterial blood flow should increase as portal blood flow decreases, and its distribution may affect the distribution of nutrients from the intestinal tract. The dual-VENC method provides two VENC settings, one for the highest velocity and the other for the lowest velocity, allowing simultaneous acquisitions of high-velocity and low-velocity components of the flow in the same FOV and in a single imaging session [50–52]. The data acquired with higher VENC may be used for assessing arterial and those acquired with lower VENC for portal venous velocities, potentially allowing evaluation of hemodynamic changes in the artery and portal vein that could not be investigated in this study. Fifth, we used a relatively low VENC of 20 cm/s to assess portal system hemodynamics. Therefore, we cannot exclude the possibility that the dataset contained cases of velocity aliasing in the highest velocity section. Finally, we did not measure the small hepatic branch of the PV in this study. The atrophy–hypertrophy complex shows atrophy in the right and left medial lobes and compensatory enlargement in the left lateral and caudate lobes [5, 12, 53, 54]. Therefore, these results should be confirmed in future clinical trials with a detailed evaluation, including intrahepatic PV branches and hepatic veins, and many patients stratified by the etiology of chronic liver disease. However, since image voxel size limits vascular evaluation, improved MRI specifications and techniques may be required for branch evaluation.

In conclusion, atrophy of the right lobe of the liver and compensatory hypertrophy of the left and caudate lobes are closely related to blood flow in the portal venous system.

## Data Availability

The datasets generated and/or analyzed during the current study are available from the corresponding author on reasonable request.
